# Delivering Remote Measurement-Based Care in Community Addiction Treatment: Engagement and Usability Over a 6-Month Clinical Pilot

**DOI:** 10.3389/fpsyt.2022.840409

**Published:** 2022-04-07

**Authors:** Kevin A. Hallgren, Eliza B. Cohn, Richard K. Ries, David C. Atkins

**Affiliations:** Department of Psychiatry and Behavioral Sciences, University of Washington, Seattle, WA, United States

**Keywords:** addiction, measurement-based care (MBC), recovery, routine outcome monitoring (ROM), user-centered design (UCD)

## Abstract

**Objective:**

Measurement-based care (MBC) is an evidence-based practice in which patients routinely complete standardized measures throughout treatment to help monitor clinical progress and inform clinical decision-making. Despite its potential benefits, MBC is rarely used in community-based substance use disorder (SUD) treatment. In this pilot study, we evaluated the feasibility of incorporating a digital and remotely delivered MBC system into SUD treatment within a community setting by characterizing patients’ and clinicians’ engagement with and usability ratings toward the MBC system that was piloted.

**Methods:**

A pilot study was conducted with 30 patients receiving SUD treatment and eight clinicians providing SUD treatment in a large, publicly funded addiction and mental health treatment clinic. Services as usual within the clinic included individual psychotherapy, case management, group therapy, peer support, and medication management for mental health and SUD, including buprenorphine. Patients who enrolled in the pilot continued to receive services as usual and were automatically sent links to complete a 22-item questionnaire, called *the weekly check-in*, *via* text message or email weekly for 24 weeks. Results of the weekly check-in were summarized on a clinician-facing web-based dashboard. Engagement was characterized by calculating the mean number of weekly check-ins completed by patients and the mean number times clinicians logged into the MBC system. Ratings of the MBC system’s usability and clinical utility were provided by patients and clinicians.

**Results:**

Patient participants (53.3% male, 56.7% white, 90% Medicaid enrolled) completed a mean of 20.60 weekly check-ins (i.e., 85.8% of the 24 expected per patient). All but one participating clinician with a patient enrolled in the study logged into the clinician-facing dashboard at least once, with an average of 12.20 logins per clinician. Patient and clinician ratings of usability and clinical utility were favorable: most patients agreed with statements that the weekly check-in was easy to navigate and aided self-reflection. All clinicians who completed usability questionnaires agreed with statements indicating that the dashboard was easy to navigate and that it provided meaningful information for SUD treatment.

**Conclusions:**

A digital and remotely delivered MBC system can yield high rates of patient and clinician engagement and high ratings of usability and clinical utility when added into SUD treatment as usual. The success of this clinical pilot may be attributable, in part, to the user-centered design processes that were used to develop and refine the MBC system that was piloted. Future efforts may focus on strategies to test whether MBC can be sustainably implemented and offers clinical benefits to patients in community SUD treatment settings.

## Introduction

Over 2.5 million US adults engage in treatment for substance use disorder (SUD) annually (1), each of whom experiences a unique clinical course and outcome. *Measurement-based care* (MBC) is a clinical method in which clinicians routinely administer standardized measures to systematically monitor their patients’ responsiveness to treatments over time and inform clinical decision-making (2–4). MBC has been tested many times in non-SUD mental health treatment settings, where it is associated with several benefits, including larger treatment effect sizes (3) and a better ability for clinicians to detect non-improvement and adjust treatment approaches accordingly (5). As a result of these findings, a growing body of research has aimed to improve the implementation of MBC in mental health treatment settings (3). Research testing the use of MBC in specialty SUD treatment settings has been limited (6, 7), even though it is possible that the benefits of MBC observed in mental health treatment settings could be extendable to SUD treatment settings. SUD treatment settings often have unique workflows, treatment approaches, clinician training requirements, and patient populations compared to non-SUD mental health treatment settings, warranting research on the development and testing of MBC systems specific to the context of SUD treatment settings.

In a previous effort to inform the design of a MBC system for outpatient adult community SUD treatment settings, members of our team conducted formative research in partnership with three community SUD treatment clinics. Through this collaboration, we aimed to understand clinicians’ ideas, concerns, and preferences related to the MBC system designs, workflows, and content (8). Results of that work indicated that clinicians saw several potential benefits of MBC, including opportunities for improved treatment delivery, patient self-reflection, and communication between patients and their providers about clinical progress. Clinicians noted that MBC systems would be particularly helpful in their settings if they (a) include options for personalization to individual patients (e.g., include questions about patients’ goals when asking about their progress, include questions that allow open-ended/free-text responses), (b) minimize burden to clinicians and patients (e.g., use technology to automatically administer and score questionnaire results, utilize patients’ smartphones rather than adding devices to clinic waiting areas, allow clinicians to access MBC results using existing their organization’s existing login credentials), and (c) measure clinical domains that reflect positive outcomes that clinicians often directly target in SUD treatments (e.g., self-efficacy, use of positive coping skills, and engagement in valued activities) as opposed to exclusively measuring negative outcomes that patients often feel stigmatized when reporting (e.g., substance use and relapse).

Informed by these perspectives, we developed a prototype of a MBC system intended for use in outpatient adult community SUD treatment settings. Following a user-centered design framework (9), the prototype was iteratively refined based on five rounds of usability testing with feedback from patients and clinicians in a large community-based SUD treatment clinic (10). This work resulted in a fully functional MBC system with two primary components: a patient-facing MBC questionnaire, called *the weekly check-in*, and a web-based clinician-facing dashboard for reviewing MBC results, called *the clinician dashboard*. The current pilot study evaluated the feasibility of using this MBC system when it is added onto SUD treatment as usual for up to 6 months. In this paper, we report outcomes related to clinicians’ and patients’ engagement with the MBC system and their assessments of its usability and clinical utility when used in conjunction with SUD treatment as usual.

## Materials and Methods

### Setting and Participants

All study procedures were approved by the University of Washington Institutional Review Board. Clinician and patient participants were recruited from two treatment teams within a large, publicly funded addiction and mental health treatment clinic owned by King County in Washington State and managed by the University of Washington. Services available in the clinic included individual psychotherapy, case management, group therapy, peer support, and medication management for mental health and SUD (including buprenorphine). Clinician participants were recruited through verbal announcements at team meetings and invitation letters placed in staff mailboxes. Clinicians who expressed interest in participating were given more information about study procedures and provided written informed consent to participate.

Patients were recruited using flyers posted in clinic waiting areas and paper handouts that participating clinicians could distribute to their patients. Patient eligibility criteria included: receiving treatment for SUD from a clinician who was also participating in the study, having a smartphone, self-reporting speaking and reading English, ≥18 years old, and reporting past year unhealthy alcohol use [measured by an Alcohol Use Disorders Identification Test-Consumption version (AUDIT-C) score ≥3 or 4 for women or men, respectively (11, 12)] and/or past-year use of illicit or non-prescribed drugs (13). Patients were ineligible if they anticipated leaving the region or becoming incarcerated within the next 6 months. Patients who were interested in participating called the study phone number listed on the flyer or handout and completed a brief eligibility screen during the phone call. Eligible participants then completed a baseline appointment, described below. The recruitment period was October 2019–June 2021, with a pause in recruitment between March and June 2020 to accommodate necessary protocol changes due to the emerging COVID-19 pandemic.

### Procedures

**FIGURE 1:**
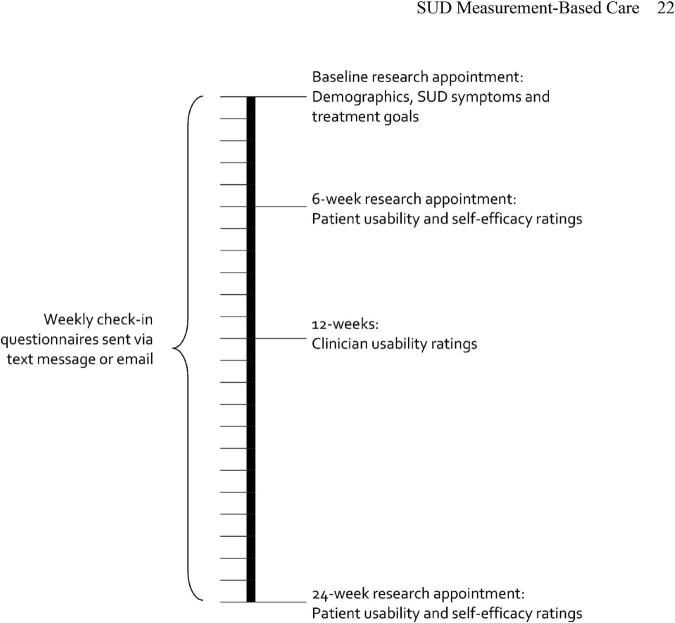
Study timeline.

Eligible patients attended a baseline appointment with a research coordinator in-person or by phone to provide informed consent for all study procedures and to complete research assessments. Patient participants met with the research coordinator again at 6-, 12-, and 24-week follow-ups to complete research assessments and structured interviews (described below). Patients received $50 for each research appointment they completed but were not compensated for completing weekly check-ins. A visual timeline for patient participants is shown in Figure 1. Clinicians received no compensation for participating.

At baseline, patient participants were non-randomly assigned to one of two conditions based on the time they enrolled in the study. Patients who enrolled between October 2019 and February 2020 were assigned to the “weekly check-in only” condition, where they completed weekly check-in questionnaires (described below), but their clinicians did not have access to the results of the weekly check-in. The purpose of this condition was to ensure that all research protocols and technologies were fully functional and acceptable before providers accessed the results to MBC questionnaires that would potentially impact patient care. Specifically, we utilized this condition to ensure that most patients were able to complete the weekly check-in and provide opportunity for them to report on its usability. During this phase of the study, we also ensured that the clinician dashboard was correctly displaying patients’ responses to the weekly check-in questionnaire. Patients who enrolled between July 2020 and June 2021 were assigned to a “weekly check-in + clinician dashboard” condition, in which patients completed weekly check-ins and the results of those weekly check-ins were accessible to clinicians through the clinician dashboard (described below). Participants in both conditions were informed as to whether their weekly check-in results would be viewable to their clinicians.

#### Patient Weekly Check-In

**FIGURE 2:**
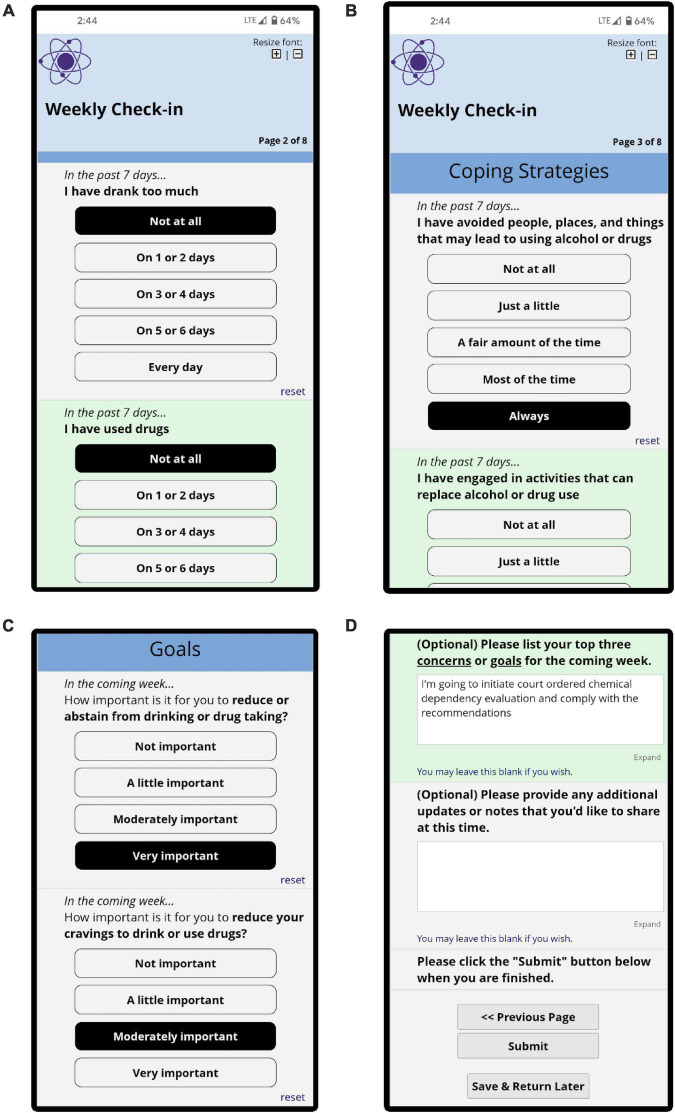
Screenshots showing selected sections of the weekly check-in completed by patients, including questions about substance use **(A)**, mechanisms of change **(B)**, next-week goals **(C)**, and optional open-ended/free-text questions **(D)**.

All patient participants were sent weekly invitations to complete a brief questionnaire called *the weekly check-in* (see Figure 2). Invitations were sent automatically each week *via* text message or email (based on patient preference) using REDCap software (14). The first weekly check-in was completed during the baseline research appointment with a research coordinator present in-person or by phone who encouraged patients to ask for assistance or clarification when needed. For patients in the weekly check-in+ clinician dashboard condition, the research coordinator encouraged patients to answer weekly check-in questionnaires with the understanding that their clinician would review their responses. When patients asked how to interpret potentially ambiguous items on the weekly check-in (e.g., whether using a specific substance counted as “drug use”), they were encouraged to answer in a way that would be most meaningful to them and most useful for communicating about their treatment progress with their clinician. At the baseline appointments, the research coordinator encouraged patients to complete weekly check-ins as early and as often as possible. The research coordinator monitored weekly check-in completion throughout the 24-week study period, and during the first 12 weeks of the study the research coordinator contacted patients to offer support completing weekly check-ins if they were not completed.

The weekly check-in assessed 8 clinical domains using 22 questions derived from existing assessment instruments (15–19). Two clinical domains asked about past-week drinking and other drug use (Figure 2A). Six clinical domains assessed areas that reflect hypothesized mechanisms of change in SUD treatment and were previously identified by SUD treatment clinicians as particularly helpful to measure as part of MBC, including past-week experiences with craving, coping skills, abstinence self-efficacy, depression symptoms, positive outlook on life, and therapeutic alliance [Figure 2B; see also (8)]. Six questions asked about goals for the upcoming week with respect to reducing substance use, reducing cravings, learning more effective coping skills, increasing abstinence self-efficacy, working on mental health, and having a more positive outlook on life (Figure 2C). Two optional questions invited patients to provide open-ended/free-text narratives describing additional goals for the upcoming week and additional information that they may wish to relay to their clinician (Figure 2D).

#### Clinician Dashboard

**FIGURE 3:**
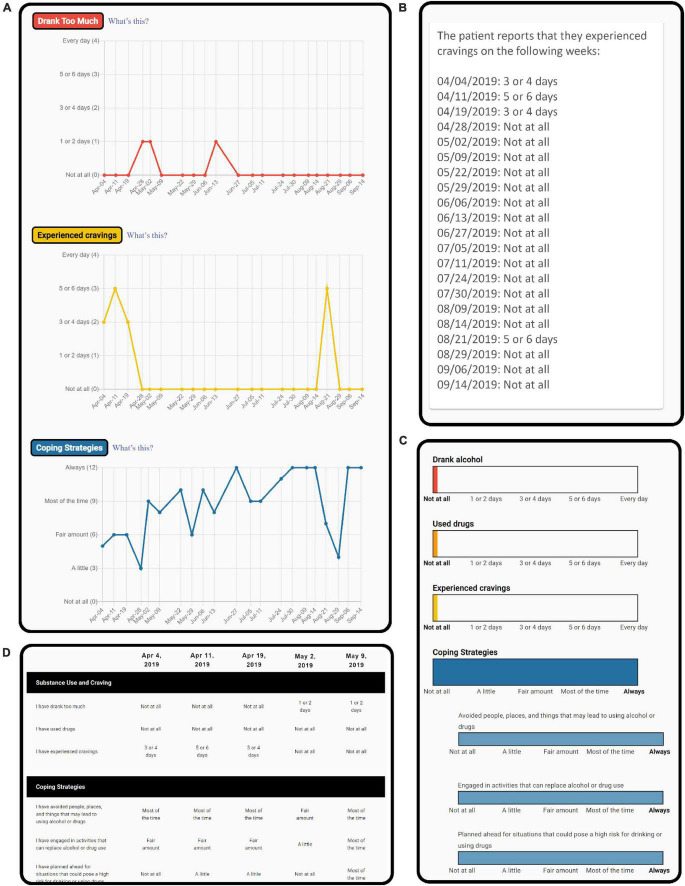
Screenshots showing selected sections of the clinician dashboard, including sections that display line graphs of patient progress over time **(A)**, text-based information about patient progress over time **(B)**, responses to the most recently completed weekly check-in **(C)**, and a table with all responses weekly check-ins previously completed **(D)**.

Clinician participants were given access to a secure web-based dashboard on which they could review summarized results from the weekly check-ins completed by patients in the weekly check-in + clinician dashboard condition. The dashboard displayed line graphs to illustrate change over time for each domain measured by the weekly check-in (Figure 3A), text-based summaries of changes in domains over time (Figure 3B) bar graphs showing the most recent responses to each question (Figure 3C), and a table displaying answers to all questions from previous weekly check-ins (Figure 3D). Clinicians received email reminders to review the dashboard every 2 weeks while they had patients enrolled in the study.

### Measures

#### Demographics

Patients completed a questionnaire to self-report their age, gender, race, ethnicity, highest level of education, marital status, employment, annual income, housing status, insurance, and current legal involvement. Clinicians completed a brief questionnaire to report their age, gender, race, ethnicity, highest education, number of years working in the current clinical setting, and typically used treatment approaches.

#### Substance Use Disorder Symptoms and Treatment Goals

Patients self-reported which substances they were addressing in treatment and completed symptom checklists (20, 21) on which they self-reported the presence or absence of each of the 11 SUD criteria for those substances, as defined by the Diagnostic and Statistical Manual, 5th Edition (DSM-5) (22). SUD severity was then categorized based on the number of symptoms reported at baseline, categorized as severe SUD (6–11 symptoms), moderate SUD (4–5 symptoms), mild SUD (2–3 symptoms), or no SUD (0–1 symptoms). Patients also self-reported whether they had a goal of abstinence, reduced use, or no specific goal to change their use of alcohol and other drugs.

#### Engagement

Patient engagement was characterized using data automatically recorded when weekly check-ins were completed. The primary engagement metrics included the mean number of weekly check-ins completed per patient over the full 24-week study period as well as the mean number of weekly check-ins completed per patient during weeks 1–12 (when the research coordinator proactively contacted patients when they did not complete the weekly check-in) and weeks 13–24 (when the research coordinator would not contact patients). We also calculated the number of weekly check-ins in which patients provided a written response to either of the two optional, open-ended questions that asked about additional goals or additional information the patient would like to relay to their clinician. We estimated the mean length of time it took to complete each weekly check-in by computing differences in timestamps for when the weekly check-in was first opened and when it was submitted, excluding durations that appeared unrealistically long (>30 min; 6.3% of weekly check-ins) as these likely reflected times when patients completed the weekly check-in over two or more sittings (i.e., patients could partially complete the weekly check-in and return to it later).

Clinician engagement with the dashboard was characterized using login and page-visit data that was automatically recorded upon logging into the clinician dashboard. We identified the number of clinicians who logged into the dashboard at least once, the mean number of dashboard logins per clinician, and the mean duration that the dashboard remained open per login session.

#### Usability and Clinical Utility

Usability and clinical utility of the weekly check-in was self-reported by patients at research appointments. On the usability questionnaire, patients were asked to rate their level of agreement with several statements about the usability of the weekly check-in (example item: “I can easily find my way on the weekly check-in”) and the clinical utility of the weekly check-in (example item: “The weekly check-in can help me reflect on what I want”). Response options for these questions were on a 5-point Likert scale ranging from strongly disagree to strongly agree. An additional question asked patients to report whether the length of the weekly check-in survey was too long, too short, or “just right.”

Patients also completed a 5-item questionnaire asking about their confidence in their ability to complete weekly check-ins independently or during a session with their clinician. Response options were on a 5-point Likert scale ranging from strongly disagree to strongly agree.

Clinicians were invited to complete a questionnaire rating their experiences using the dashboard with each of their patients in the weekly check-in + clinician dashboard condition. The questionnaire included items about the dashboard’s usability (example item: “I could easily find my way on the dashboard”) and clinical utility (example item: “The dashboard provided me with useful information”). Response options were on a 5-point Likert scale ranging from strongly disagree to strongly agree. The questionnaire was emailed to clinicians approximately 12 weeks after their patient enrolled in the study.

### Analysis Plan

Descriptive statistics were used to characterize patients’ SUD severity, substance use goals, and the measures of patient and clinician demographics, engagement, and usability that were described above. Rates of weekly check-in completion across the 24-week study period for all patients were estimated overall and by study period (weeks 1–12, weeks 13–24) and for each study week. Questionnaire results for the usability and clinical utility of the weekly check-in were analyzed descriptively for the 6- and 24-week time points to understand perceptions of usability and clinical utility earlier and later in the span of using the weekly check-in.

## Results

### Description of Samples

Sixty-three individuals called the study phone number to inquire about participating in the study. Sixty-two completed the eligibility screening, of which 33 were eligible to participate, 28 were ineligible, and 1 was eligible but declined to participate. Of the 28 ineligible individuals, 21 were ineligible because they were not receiving care in the participating clinic and/or receiving care from a participating clinician, and 7 were ineligible because they did not report any past-year unhealthy alcohol use or any past-year drug use. A total of 30 patients completed a baseline enrolled appointment, including 16 in the weekly check-in only condition and 14 in the weekly check-in + clinician dashboard condition. Patient participants are described in Table 1. The distributions of age, gender, race, and ethnicity were similar to that of the full clinic population, according to electronic health care record data from the clinic. Most patients were aged 35–54 (*n* = 19), male (*n* = 16), and white (*n* = 17) and most had an associate’s degree, trade degree, or other higher education degree (*n* = 23). Most patients were not currently employed (*n* = 27), just over half (*n* = 16) were homeless, in transitional, temporary, or other housing, or living in a house someone else owned or leased. Most reported symptoms consistent with severe SUD (*n* = 23). Patients reported that their treatment was addressing use of stimulants (*n* = 18), opioids (*n* = 16), alcohol (*n* = 15), cannabis (*n* = 5), sedatives (*n* = 4), and hallucinogens (*n* = 1). With regard to alcohol, patients reported goals of abstinence (*n* = 12), reduced drinking (*n* = 5), or had no specific goal for changing alcohol use (*n* = 13). With regard to other drugs, patients reported goals of abstinence (*n* = 16), reduced use (*n* = 5), or had no specific goal for changing drug use (*n* = 9). All 30 patients completed the baseline and 6-week research appointments; 29 patients completed the 12- and 24-week research appointments.

**TABLE 1:** Characteristics of patient participants (*N* = 30).

	*n*	(%)
**Age**		
25–34	8	(26.7%)
35–44	9	(30.0%)
45–54	10	(33.3%)
55–65	3	(10.0%)
**Gender**		
Female	11	(36.7%)
Male	16	(53.3%)
Non-binary	2	(6.7%)
Prefer not to say	1	(3.3%)
**Race**		
American Indian or Alaska Native	2	(6.7%)
Asian	1	(3.3%)
Black or African American	4	(13.3%)
Native Hawaiian or Pacific Islander	0	(0.0%)
White or Caucasian	17	(56.7%)
Another race not listed	6	(20.0%)
**Hispanic or Latino (any race)**	2	(6.7%)
**Highest education**		
Less than high school	1	(3.3%)
High school diploma or equivalent	6	(20.0%)
Some college, associate’s degree, or trade degree	17	(56.7%)
Bachelor’s degree or higher	6	(20.0%)
**Employed currently (part time or full time)**	3	(10.0%)
**Income below federal poverty level for single person household**	17	(56.7%)
**Housing**		
In a home owned or leased by participant	14	(46.7%)
In a home someone else owns or leases	8	(26.7%)
Transitional, temporary, other housing, or homeless	8	(26.7%)
**Married or in a committed relationship**	6	(20.0%)
**Medicaid enrolled**	27	(90.0%)
Current legal system involvement*	5	(16.7%)
**SUD symptoms, past year**		
0–1	3	(10.0%)
2–3 (mild SUD)	4	(13.3%)
4–5 (moderate SUD)	0	(0.0%)
6+ (severe SUD)	23	(76.7%)

*Current legal system involvement including drug court, probation, parole, current legal charges, house arrest, court-mandated treatment, or awaiting sentencing.

Eight clinicians enrolled in the study. Their age, gender, race/ethnicity, education, experience working in the clinical setting, and treatment approaches are described in Table 2. Seven clinicians had at least 1 patient enroll in the study (median = 4 patients per clinician, range: 1–8). Six clinicians had at least 1 patient enroll in the weekly check-in + clinician dashboard condition (median = 2 patients per clinician, range: 1–4), and thus these six clinicians were able to review their patients’ progress on the clinician dashboard and were invited to the complete dashboard usability questionnaire.

**TABLE 2:** Characteristics of clinician participants (*n* = 8).

Clinicians (*N* = 8)	*N*	(%)
**Age**		
25–44	3	(37.5%)
45–64	5	(62.5%)
**Gender**		
Female	3	(37.5%)
Male	5	(62.5%)
**Race and ethnicity**		
Black or African American, non-Hispanic	1	(12.5%)
White or Caucasian, non-Hispanic	7	(87.5%)
**Highest education**		
Bachelor’s degree	2	(25.0%)
Master’s degree	6	(75.0%)
**Number of years worked in the current clinical setting, median (range)**	5	(2 to 18)
**Clinical approaches used**		
Case management	7	(87.5%)
Client-centered/humanistic counseling	5	(62.5%)
Cognitive-behavioral therapy	4	(50.0%)
Family or couples therapy	1	(12.5%)
Motivational interviewing	5	(62.5%)
Twelve-step based treatment	2	(25.0%)
Psychodynamic/psychoanalytic	2	(25.0%)
Relapse prevention	5	(62.5%)
Medication management	2	(25.0%)
Other approaches	2	(25.0%)
		

### Patient and Clinician Engagement

**FIGURE 4:**
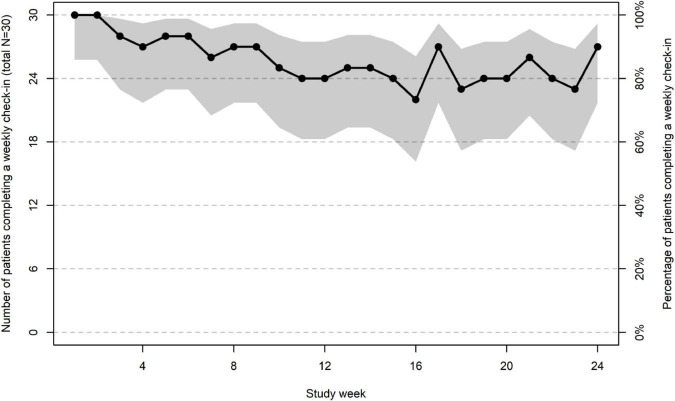
Number **(left axis)** and percentage **(right axis)** of patients completing a weekly check-in during each week of the clinical pilot. The shaded region reflects the 95% CI of the estimated percentage for each week.

Twenty-nine patients elected to receive weekly check-in prompts *via* text message and one elected to receive them *via* email. Patient engagement metrics are described in the upper half of Table 3. Rates of weekly check-in completion for all patients over the 24-week pilot are shown in Figure 4. On average, patient participants completed 20.60 weekly check-ins (85.8% of the 24 available to each patient). All patients completed the first 2 weekly check-ins, and the proportion of patients completing the weekly check-in decreased slightly over time until week 12, at which time 80% of patients completed the weekly check-in (Figure 4). Between weeks 13–24, rates of weekly check-in completion remained stable with approximately 80% of patients completing it each week (Figure 4). Patients provided a write-in response to either or both of the optional, open-ended questions on a mean of 9.17 (*SD* = 7.90) of the weekly check-ins that were completed (44.5% of completed weekly check-ins). Patients in the weekly check-in only condition and the weekly check-in + clinician dashboard condition did not differ in the number of weekly check-ins completed (*p* = 0.33) or the number of weekly check-ins with a write-in response (*p* = 0.94). The mean estimated time to complete each weekly check-in was 4.99 min (*SD* = 4.46).

**TABLE 3:** Patient and clinician engagement metrics.

Patient engagement (*N* = 30)	M	(SD)
Number of weekly check-ins completed per patient (full 24-week period)	20.60	(5.54)
Number of weekly check-ins completed per patient (weeks 1–12)	10.80	(2.23)
Number of weekly check-ins completed per patient (weeks 13–24)	9.80	(3.46)
Number of weekly check-ins with an open-text response, per patient	9.17	(7.90)
Time to complete each weekly check-in (min.)*^a^*	4.99	(4.46)
**Clinician engagement (*N* = 5 clinicians with ≥ 1 patient in weekly check-in + clinician dashboard condition who logged into the clinician dashboard)**		
Number of dashboard login sessions per clinician	12.20	(9.33)
Time spent using dashboard per login session (min.)	2.30	(4.61)

*^a^Estimated based on the difference in time between when the weekly check-in was first opened and when it was submitted.*

Five out of six clinicians who had a patient in the weekly check-in + clinician dashboard condition logged into the clinician dashboard at least once. Among them, there was a mean of 12.20 logins per clinician (*SD* = 9.33, range = 3–25). On average, each login session lasted 2.30 min.

### Usability and Clinical Utility

Usability ratings were favorable at the 6- and 24-week time points (Table 4), with most patients (86.2–100%) agreeing or strongly agreeing with statements that the weekly check-in was helpful for reflecting on their substance and recovery, that they would be willing to use the weekly check-in in the future, and that they would recommend the weekly check-in to others. Most patients (86.2%) described the length of the weekly check-in as “just right” at both time points. Most patients also reported feeling confident in their ability to complete weekly check-ins independently and/or during treatment sessions (82.8–100%) and few reported that they would feel stress completing weekly check-ins independently or during treatment sessions (6.9–14.3%).

**TABLE 4:** Patient ratings of usability, clinical utility, and self-efficacy completing weekly check-ins.

	Week 6 (*n* = 29)					Week 24 (*n* = 29)				
		
Usability and clinical utility of the weekly check-in	Strongly disagree	Disagree	Neutral	Agree	Strongly agree	Strongly disagree	Disagree	Neutral	Agree	Strongly agree
I can easily find my way on the weekly check-in.	1		1	2	24	1			4	23
I am satisfied with the language used on the weekly check-in.			2	8	19			1	8	20
The weekly check-in survey is interesting.	1	1	8	13	5		2	9	8	9
The weekly check-in survey does not contain distracting elements.		1	1	8	17			3	7	18
I find the weekly check-in helpful.		2	2	13	12		1	3	9	16
The weekly check-in can help me reflect on what I want.		1	2	14	12				12	17
The weekly check-in helps me reflect on my substance use and recovery.			1	10	18				10	19
I can imagine myself discussing the information on the weekly check-in with my clinician.	1	4	6	7	11			10	7	12
I can imagine the weekly check-in being helpful to others.			2	9	18			1	12	16
I would be willing to use the weekly check-in in the future.		1		11	17		1	3	5	20
I would recommend the weekly check-in to others.		1	2	11	15			2	8	19

**Length of the weekly check-in**	**Much too short**	**Too short**	**About right**	**Too long**	**Much too long**	**Much too short**	**Too short**	**About right**	**Too long**	**Much too long**

The length of the weekly check-in is:		1	25	3			3	25	1	

	**Week 6 (*n* = 29)**					**Week 24 (*n* = 28)**				
		
**Self-efficacy for completing the weekly check-in**	**Strongly disagree**	**Disagree**	**Neutral**	**Agree**	**Strongly agree**	**Strongly disagree**	**Disagree**	**Neutral**	**Agree**	**Strongly agree**

I have been able to understand the questions that were asked in the weekly check-in.	1			7	21				5	23
I would feel confident in my ability to answer similar questions if I were completing the weekly check-in at home by myself.	1	1		6	21				6	22
I would feel confident in my ability to answer similar questions if I were completing the weekly check-in during my treatment.		1	3	5	19				8	20
I would feel stressed if I were asked to complete the weekly check-in while I was at home by myself.	15	10	1	1	2	18	5	2	1	2
I would feel stressed if I were asked to complete the weekly check-in while I was in a treatment session with my clinician.	12	8	6	2		14	7	3	3	1

Five out of six clinicians with patients in the weekly check-in + clinician dashboard condition completed a dashboard usability questionnaire (mean = 2 questionnaires per clinician). Usability and clinical utility ratings were favorable (Table 5), with all clinicians reporting that the dashboard was easy to navigate, that the information was meaningful and could be helpful to clinicians who offer alcohol or drug treatment, and that they would be willing to use the dashboard in the future. Most clinicians also said that they would be able to use the dashboard during sessions with patients and that the information included on it was helpful to their patients.

**TABLE 5:** Clinician ratings of usability and clinical utility (*n* = 5)*.

Usability and clinical utility of the clinician dashboard	Strongly disagree	Disagree	Neutral	Agree	Strongly agree
I could easily find my way on the dashboard.				1	4
I was satisfied with the language used on the dashboard.				2	3
The dashboard provided me with meaningful information.				3	2
The information on the dashboard was helpful to my patient.			1	2	1
The information on the dashboard can be helpful to clinicians who offer alcohol or drug treatment.				2	3
I was able to discuss the information on the dashboard with my patient.				3	
I would be willing to use the dashboard in the future.				1	4
I would be able to use the dashboard during sessions with patients.				3	2

*Clinicians could complete a usability questionnaire for each patient they had enrolled in the weekly check-in + dashboard condition. When a participant completed multiple questionnaires, the average ratings across questionnaires for that participant were used.

## Discussion

Results from this clinical pilot provide preliminary support for the feasibility of incorporating a digital, remotely delivered MBC system into SUD treatment as usual in a community SUD treatment setting. Among patients and clinicians who consented to participate in this 6-month pilot, rates of engagement with the MBC system were high for patients (e.g., patients completed 85.8% of weekly check-ins, with optional free-text responses included in 44.5% of the weekly check-ins that were completed) and for clinicians (e.g., clinicians logged into the dashboard a mean of 12.20 times). Further, the system seemed to impose minimal time burden to patients and clinicians, who on average took less than 5 min to complete weekly check-ins and less than 3 min to review MBC results on the clinician dashboard, respectively. Usability ratings were favorable, with most patients reporting that the weekly check-in was interesting, helpful for self-reflection, and something they would be willing to continue using, and most clinicians reporting that the information on the dashboard was helpful and that they discussed the information with their patient.

While SUD treatment providers have been previously shown to report positive attitudes toward MBC (8, 23), studies have also identified numerous barriers that can impede the implementation of MBC in SUD treatment settings (5, 23–25). Notably, implementation barriers may vary between different SUD treatment settings and may be associated with patient-, provider-, and system-level factors (23). For example, patients may have difficulty completing measures due to illness, disability, or distress; they may perceive completing MBC questionnaires as not being personally meaningful or useful; and they may experience difficulty using technology or experience usability-related barriers (24). Clinicians may perceive information provided by MBC as impersonalized or unreliable; they may experience additional workload associated with administering, scoring, and reviewing measures; and they may feel uncomfortable or uncertain about how to integrate MBC into their clinical practice (8, 23). Healthcare systems may also lack adequate structures to support MBC due to a lack of training and technical support for MBC; payment models that do not reimburse for time spent using MBC; and limited integration of MBC systems with other technologies that are used by patients and clinicians (25, 26).

Despite numerous potential barriers, the current pilot study found that digital, remotely delivered MBC was feasible to incorporate into SUD treatment as usual with high rates of engagement and high ratings of usability and clinical utility reported by patients and clinicians. It is possible that the positive findings obtained here are partly attributable to the user-centered design methods that informed the specific designs, workflows, and contents included in the MBC system that was piloted in the current study (8, 10). For example, informed by stakeholder input, we designed the MBC system to allow patients to complete weekly check-ins on their personal smartphones (in contrast to our initial idea of using tablet computers or paper questionnaires in clinic waiting rooms), which may address some implementation barriers cited above by integrating the weekly check-in with existing technologies used by patients and by eliminating the need for clinicians to administer and score MBC measures. The user-centered design approach also led us to measure domains that clinicians identified as most clinically helpful, to include questions about patients’ treatment goals, and to include open-ended questions that invited optional free-text responses, potentially reducing barriers related potential lack of personalization in MBC. Many of the clinical domains in the weekly check-in reflected positive outcomes (e.g., self-efficacy, use of coping skills, and positive outlook on life) rather than focusing more exclusively on outcomes that are perceived as negative or stigmatized (e.g., substance use, relapse, and SUD symptoms), potentially helping patients and clinicians reflect on positive experiences and growth, rather than focusing more exclusively on negative experiences (e.g., substance use and SUD symptoms). Usability testing also helped us iteratively improve the format of the weekly check-in (e.g., optimizing the layout for mobile devices, improving the consistency of wording used in questions and response options) and the clinician dashboard (e.g., displaying results graphically and in text formats), potentially reducing usability-related barriers.

Seeking and incorporating input from clinicians and patients in SUD treatment settings may be critical to high rates of engagement, and adequate usability of MBC systems, which in turn may be key facilitators to implementing MBC into routine care (27). Findings from another study are consistent with this emphasis on stakeholder engagement; for example, in recent pilot study, Russell and colleagues (28) successfully pilot tested the use of a MBC system in a 15-bed residential adolescent SUD treatment setting after conducting multiple rounds of stakeholder engagement and collaborative development of MBC workflows and questionnaire items. Developing clinical technologies through user-centered design approaches may be necessary for producing clinical technologies that are more usable, engaging, and sustainable in clinical settings (29). In addition, this user-centered design approach honors the lived experiences and expertise patients and clinicians in SUD treatment settings and helps their voices be heard in clinical research and technology development.

Delivering MBC using digital technologies that are accessible from any location (including outside of the clinic) may provide several advantages in SUD treatment settings that may have also contributed to the high engagement and usability observed in this study (30). For example, patients can continue to complete weekly check-ins from any location, including when they might have irregular or infrequent contact with the clinic. This might occur when scheduled treatment sessions are scheduled infrequently, conducted virtually, missed, or inaccessible due to barriers to attendance (e.g., difficulty traveling to clinic and COVID-19-related restrictions). Digital platforms also could potentially help minimize burden to clinicians by allowing weekly check-in reminders to be automatically sent to patients and for the data in the weekly check-ins to be automatically scored, stored, and presented on the clinician dashboard in numerous formats.

Measuring potential mechanisms of change in SUD treatment—including craving, coping skills, abstinence self-efficacy, engagement in valued activities, depression symptoms, and therapeutic alliance—may be a valuable contrast to the common practice for outcome measures to focus on substance use as the primary treatment outcome. For example, many clinical trials of SUD treatments measure abstinence and/or reductions in substance use as the primary clinical endpoint. Likewise, in real-world clinical practice it is common for patients to discuss how long they have been abstinent from alcohol and/or drugs or how often they use substances to gauge their treatment progress. However, gauging substance use treatment progress by focusing primarily on substance use and abstinence may reinforce existing stigma and black-and-white thinking related to substance use (e.g., a person is either succeeding or failing based on whether they are drinking or using drugs), while also failing to capture a more complete or holistic understanding of patient progress across a range of clinical domains during treatment. In contrast, assessing multiple clinical domains, including measures that do not directly reflect substance use or abstinence, may help patients and clinicians better understand treatment progress more holistically. It also may potentially deemphasize abstinence or reduced substance use as the sole purpose of SUD treatment and instead help emphasize that SUD treatment can potentially impact multiple dimensions within a person’s life.

There are important limitations to this study. The MBC system and procedures were tested within a single, large, publicly funded addiction and mental health treatment program affiliated with an academic medical center; therefore, results may not generalize to other types of settings. By design, we recruited a small sample for this pilot study, which precluded us from conducting subgroup analyses that could evaluate whether engagement, usability, and clinical utility ratings varied between specific subgroups. Our sample of clinicians also was small and predominantly white and non-Hispanic. The sample only included patients with smartphones, and while most patients in SUD treatment have smartphones (31, 32), the approaches used here would not be accessible to all patients in SUD treatment. All patients in the sample elected to participate in a research study focusing on MBC, received payments for attending research interviews (but not for completing MBC questionnaires), and were supported by a research coordinator for the first 3 months of the 6-month trial, and thus the results may not fully capture feasibility and engagement of the MBC system if it were implemented for all patients outside of a research study context. The clinical domains that were assessed in the weekly check-in were informed by the preferences of clinicians from the same setting; however, the clinical utility of these domains could vary across treatment settings and other measures that have been proposed for MBC in SUD treatment were not tested here, such as the Brief Addiction Monitor (33–35), the Outcome Questionnaire-45 (36, 37), or measures based on SUD symptoms (20, 21). Finally, research testing the impact of MBC on patients’ clinical outcomes in SUD treatment has been limited to date (6, 7), although one study has suggested that MBC may help some patients in SUD treatment make faster reductions in their alcohol use (36).

There are also important strengths of this study. The MBC system was tested within a community treatment setting added onto treatment as usual, bolstering the external validity of the findings. Patients in the sample were diverse with respect to age, gender, race, education, and housing and reflected the demographic distribution of patients within the clinic. Clinicians in the study reported using multiple types of treatment approaches, suggesting that engagement and usability results are not contingent on providers using a specific treatment modality. Data on engagement, usability, and clinical utility were obtained from multiple modalities (automatically generated engagement measures and self-report usability measures) and from both clinician and patient participants, providing multiple perspectives about the reactions to the MBC system that was tested.

## Conclusion

Results from this clinical pilot suggest that the MBC system tested here can potentially be feasibly incorporated into existing SUD treatment settings with high rates of patient and clinician engagement, high usability and clinical utility, and minimal clinical disruptiveness. These findings lend support for additional efforts to test methods for implementing MBC into routine care in SUD treatment settings and to evaluate the impact of MBC on SUD treatment processes (e.g., therapeutic alliance, shared decision making, patient empowerment, and stigma reduction). Future studies should further evaluate the impact of MBC on patient outcomes, including outcomes related and unrelated to substance use (e.g., treatment engagement, goal attainment, and patient experience). Additionally, future research should evaluate strategies for implementing MBC as part of standard of care for patients in SUD treatment, including across clinical settings that offer different treatment modalities and that serve diverse patient populations who may have different requirements for successful MBC implementation.

## Data Availability Statement

The raw data supporting the conclusions of this article will be made available by the authors, without undue reservation.

## Ethics Statement

The studies involving human participants were reviewed and approved by the University of Washington Institutional Review Board. The patients/participants provided their written informed consent to participate in this study.

## Author Contributions

KH contributed to the study design, oversight, data analysis, and writing of the results. RR and DA contributed to the study design and writing of the results. EC contributed to the data collection and writing of the results. All authors contributed to the article and approved the submitted version.

## Author Disclaimer

The content is solely the responsibility of the authors and does not necessarily represent the official views of NIAAA or the NIH.

## Conflict of Interest

The authors declare that the research was conducted in the absence of any commercial or financial relationships that could be construed as a potential conflict of interest.

## Publisher’s Note

All claims expressed in this article are solely those of the authors and do not necessarily represent those of their affiliated organizations, or those of the publisher, the editors and the reviewers. Any product that may be evaluated in this article, or claim that may be made by its manufacturer, is not guaranteed or endorsed by the publisher.
