# Supplementary material for: Delivering Remote Measurement-Based Care in Community Addiction Treatment: Engagement and Usability Over a 6-Month Clinical Pilot
Source: Front Psychiatry. 2022 Apr 7;13:840409. doi: 10.3389/fpsyt.2022.840409 (PMC9021526; doi:10.3389/fpsyt.2022.840409)
Supplement: Supplementary file 1 [file Data_Sheet_1.pdf]

Questions included in the weekly check-in. The weekly check-in included questions about 8 clinical domains previously identified by clinicians as clinically useful to assess as part of MBC in SUD treatment (Tauscher et al., 2021). These domains included drinking, drug use, craving, depression, coping strategies, confidence in avoiding alcohol/drug use, positive outlook on life, and therapeutic alliance. There were 14 questions on the weekly check-in that asked about past-week experiences in each of these domains (1-3 questions per domain) and 6 questions that asked patients about their goals for the upcoming week on each domain except therapeutic alliance (goals for drinking and drug use were merged into a single question). All questions on the weekly check-in were obtained from previously developed instruments; however, only subsets of questions were used to derive shorter measures and the specific wording of questions and response options was often modified based on feedback we received in prior usability testing.

Drinking and drug use was measured using two questions that asked how many days the patient drank too much or used drugs over the past week. The items were from the Substance Use Recovery Evaluator (SURE; Neale et al., 2016).

Craving was measured using one question that asked how many days the patient experienced cravings over the past week. The item was from the SURE (Neale et al., 2016).

Depression was measured using the Patient Health Questionnaire-2 (PHQ-2) (Kroenke et al., 2003), which is a two-item measure derived from the nine-item PHQ-9 (Kroenke et al., 2001).

Coping strategies were measured using three questions asking patients how often they used coping strategies that could help them avoid using alcohol or drugs. The three items were from the Coping Strategies Scale (Litt et al., 2003).

Confidence in avoiding alcohol/drug use was measured using two items asking patients how confident they felt in their ability to not drink or use drugs (i.e., abstinence self-efficacy) when they were emotionally upset/in physical pain or when they felt an urge/craving over the past week. The questions were from the Brief Situational Confidence Questionnaire-8 (BSCQ-8; Breslin et al., 2000).

Positive outlook on life was measured using two questions asking how happy patients felt with their overall quality of life and how much they had realistic hopes and goals for themselves. The questions were from the SURE (Neale et al., 2016).

Therapeutic alliance was measured using two questions asking how much their providers agreed with them about what was important to work on in treatment and how often their providers gave them new ways of looking at their problems. The questions were from the Working Alliance Inventory-Short Revised (WAI-SR; Munder et al., 2010).

Goals were measured using six questions that asked patients about their goals for the next week on the domains above except therapeutic alliance (e.g., "In the coming week, how important is it for you to reduce your cravings to drink or use drugs?"). Response scales were modeled after the goal questions that were included in the SURE (Neale et al., 2016).

The text used in questions and response options are included below. Note, however, that during the pilot study reported on here, the questionnaire was displayed in a format that was optimized for mobile layouts.

[Introductory page]

Hi there! Please take a few moments to check in using the survey below.

The survey will ask you about your experiences and treatment goals.

Your responses will not be immediately reviewed by an individual on our research team. So <u>if you require urgent help or are experiencing an emergency, please dial 911 for immediate assistance.</u>

Remember to click the "submit" button when you are done. You may also save your responses and return later if you cannot complete the entire survey right now.

If you have any concerns or questions, please contact XXXXX at xxxx@xxxx.xxx or xxx-xxx-xxxx.

Thank you!

[Questionnaire pages]

In the past 7 days...
I have drank too much

- ◯ Not at all
- ◯ On 1 or 2 days
- ◯ On 3 or 4 days
- ◯ On 5 or 6 days
- ◯ Every day

In the past 7 days...
I have used drugs

- ◯ Not at all
- ◯ On 1 or 2 days
- ◯ On 3 or 4 days
- ◯ On 5 or 6 days
- ◯ Every day

In the past 7 days...
I have experienced cravings

- ◯ Not at all
- ◯ On 1 or 2 days
- ◯ On 3 or 4 days
- ◯ On 5 or 6 days
- ◯ Every day

Coping Strategies

In the past 7 days...
I have avoided people, places, and things that may lead to using alcohol or drugs

- ◯ Not at all
- ◯ Just a little
- ◯ A fair amount of the time
- ◯ Most of the time
- ◯ Always

In the past 7 days...
I have engaged in activities that can replace alcohol or drug use

- ◯ Not at all
- ◯ Just a little
- ◯ A fair amount of the time
- ◯ Most of the time
- ◯ Always

In the past 7 days...
I have planned ahead for situations that could pose a high risk for drinking or using drugs

- ◯ Not at all
- ◯ Just a little
- ◯ A fair amount of the time
- ◯ Most of the time
- ◯ Always

Confidence

How confident are you that you *would not* drink or use drugs if you were emotionally upset or in pain?

- ◯ Not at all confident
- ◯ Somewhat confident
- ◯ Moderately confident
- ◯ Very confident
- ◯ Extremely confident

How confident are you that you *would not* drink or use drugs if you felt an urge or craving?

- ◯ Not at all confident
- ◯ Somewhat confident
- ◯ Moderately confident
- ◯ Very confident
- ◯ Extremely confident

Outlook on Life

In the past 7 days...

I have felt happy with my overall quality of life

- ○ Not at all
- ○ Just a little
- ○ A fair amount of the time
- ○ Most of the time
- ○ Always

In the past 7 days...

I have had realistic hopes and goals for myself

- ○ Not at all
- ○ Just a little
- ○ A fair amount of the time
- ○ Most of the time
- ○ Always

Therapeutic Alliance

My providers in the Mental Health and Addictions Clinic and I agree on what is important for me to work on

- ○ Seldom
- ○ Sometimes
- ○ Fairly often
- ○ Very often
- ○ Always

My providers in the Mental Health and Addictions Clinic have given me new ways of looking at my problems

- ○ Seldom
- ○ Sometimes
- ○ Fairly of often
- ○ Very often
- ○ Always

Depression

In the past two weeks...
I have had little interest or pleasure in doing things

- ◯ Not at all
- ◯ Several days
- ◯ More than half the days
- ◯ Nearly every day

In the past two weeks...
I have felt down, depressed, or hopeless

- ◯ Not at all
- ◯ Several days
- ◯ More than half the days
- ◯ Nearly every day

Goals

In the coming week...
How important is it for you to **reduce or abstain from drinking or drug taking?**

- ◯ Not important
- ◯ A little important
- ◯ Moderately important
- ◯ Very important

In the coming week...
How important is it for you to **reduce your cravings to drink or use drugs?**

- ◯ Not important
- ◯ A little important
- ◯ Moderately important
- ◯ Very important

In the coming week...
How important is it for you to **learn more effective coping strategies?**

- ◯ Not important
- ◯ A little important
- ◯ Moderately important
- ◯ Very important

In the coming week...
How important is it for you to **become more confident in your ability *not* to drink or use drugs?**

- ◯ Not important
- ◯ A little important
- ◯ Moderately important
- ◯ Very important

In the coming week...
How important is it for you to **have a more positive outlook on life?**

- ◯ Not important
- ◯ A little important
- ◯ Moderately important
- ◯ Very important

In the coming week...
How important is it for you to **work on your mental health?**

- ◯ Not important
- ◯ A little important
- ◯ Moderately important
- ◯ Very important

(Optional) Please list your top three concerns or goals for the coming week.

You may leave this blank if you wish.

(Optional) Please provide any additional updates or notes that you'd like to share at this time.

You may leave this blank if you wish.

References

Breslin, F. C., Sobell, L. C., Sobell, M. B., & Agrawal, S. (2000). A comparison of a brief and long version of the Situational Confidence Questionnaire. *Behaviour Research and Therapy*, *38*(12), 1211–1220.

Kroenke, K., Spitzer, R. L., & Williams, J. B. (2001). The PHQ-9: Validity of a brief depression severity measure. *Journal of General Internal Medicine*, *16*(9), 606–613.

Kroenke, K., Spitzer, R. L., & Williams, J. B. W. (2003). The Patient Health Questionnaire-2: Validity of a two-item depression screener. *Medical Care*, *41*(11), 1284–1292.

Litt, M. D., Kadden, R. M., Cooney, N. L., & Kabela, E. (2003). Coping skills and treatment outcomes in cognitive-behavioral and interactional group therapy for alcoholism. *Journal of Consulting and Clinical Psychology*, *71*(1), 118.

Munder, T., Wilmers, F., Leonhart, R., Linster, H. W., & Barth, J. (2010). Working Alliance Inventory-Short Revised (WAI-SR): Psychometric properties in outpatients and inpatients. *Clinical Psychology & Psychotherapy: An International Journal of Theory & Practice*, *17*(3), 231–239.

Neale, J., Vitoratou, S., Finch, E., Lennon, P., Mitcheson, L., Panebianco, D., Rose, D., Strang, J., Wykes, T., & Marsden, J. (2016). Development and validation of “SURE”: A patient reported outcome measure (PROM) for recovery from drug and alcohol dependence. *Drug & Alcohol Dependence*, *165*, 159–167.

Tauscher, J. S., Cohn, E. B., Johnson, T. R., Diteman, K. D., Ries, R. K., Atkins, D. C., & Hallgren, K. A. (2021). What do clinicians want? Understanding frontline addiction treatment clinicians’ preferences and priorities to improve the design of measurement-based care technology. *Addiction Science & Clinical Practice*, *16*(1), 1–15.
